# The adverse effect of gestational diabetes mellitus and hypertensive disorders of pregnancy on maternal–perinatal outcomes among singleton and twin pregnancies: a retrospective cohort study (2011–2019)

**DOI:** 10.3389/fendo.2023.1267338

**Published:** 2023-11-30

**Authors:** Nawsherwan, Zhiyi Liu, Zhang Le, Sumaira Mubarik, Yanmei Sun, Shafaq Naeem, Hui Li

**Affiliations:** ^1^ Xiamen Cardiovascular Hospital of Xiamen University, School of Medicine, Xiamen University, Xiamen, China; ^2^ Clinical College of Traditional Chinese Medicine, Hubei University of Chinese Medicine, Hubei, China; ^3^ PharmacoTherapy, -Epidemiology and -Economics, Groningen Research Institute of Pharmacy, University of Groningen, Groningen, Netherlands; ^4^ Department of Epidemiology and Biostatistics, School of Public Health, Wuhan University, Wuhan, China; ^5^ Department of Obstetrics and Gynecology, Renmin Hospital, Wuhan University, Wuhan, Hubei, China; ^6^ Department of Preventive Medicine, School of Public Health, Wuhan University, Wuhan, China; ^7^ Department of Medicine, Taixing People Hospital, Taizhou, Jiangsu, China

**Keywords:** twins, singleton, gestational diabetes mellitus, hypertensive disorders of pregnancy, adverse pregnancy outcomes

## Abstract

**Background:**

Gestational diabetes mellitus (GDM) and hypertensive disorders of pregnancy (HDP) are the predominant pregnancy complications among singleton and twin pregnancies worldwide. Our primary objective was to explore the adverse effect of GDM and HDP on maternal–perinatal outcomes compared with non-GDM and non-HDP in singleton and twin pregnancies. The secondary objective was to find the risk of adverse maternal–perinatal outcomes in twin pregnancies compared with singleton pregnancies complicated with GDM and HDP in Hubei, China.

**Methods:**

A tertiary hospital-based retrospective study was conducted at Wuhan University Renmin Hospital, Hubei Province, China, from 2011 to 2019. A chi-square test was used to determine the difference in adverse maternal–perinatal outcomes between singleton and twin pregnancies. A multiple binary logistic regression model and a joinpoint regression model were used to determine the association of GDM and HDP with adverse maternal–perinatal outcomes and GDM and HDP temporal trend among singleton and twin pregnancies.

**Results:**

The trend of HDP [average annual percentage change (AAPC) 15.1% (95% confidence interval (95%CI): 5.3, 25.7)] among singleton pregnancies and GDM [AAPC 50.4% (95%CI: 19.9, 88.7)] among twin pregnancies significantly increased from 2011 to 2019. After adjusting for confounding factors, GDM is associated with an increased risk of C-section (adjusted odds ratio (aOR), 1.5; 95%CI: 1.3, 1.6) and macrosomia (aOR, 1.3; 95%CI: 1.1, 1.6) in singleton and preterm birth (PTB) (aOR, 2.1; 95%CI: 1.2, 3.3) in twin pregnancies compared with non-GDM. HDP was associated with a higher risk of C-section, PTB, perinatal mortality, and low birth weight (LBW) in both singleton and twin pregnancies compared with the non-HDP. Compared with singleton pregnancies complicated with GDM and HDP, twin pregnancies showed higher odds of C-section [(aOR, 1.7; 95%CI: 1.1, 2.7), (aOR, 4.6; 95%CI: 2.5, 8.7), respectively], PTB [(aOR, 22.9; 95%CI: 14.1, 37.3), (aOR, 8.1; 95%CI: 5.3, 12.3), respectively], LBW [(aOR, 12.1; 95%CI: 8.2, 18.1), (aOR, 5.1; 95%CI: 3.6, 7.4), respectively], and low Apgar score [(aOR, 8.2; 95%CI: 4.4, 15.1), (aOR, 3.8; 95%CI: 2.4, 5.8), respectively] complicated with GDM and HDP.

**Conclusion:**

In conclusion, GDM showed an increased risk of a few adverse maternal–perinatal outcomes and HDP is associated with a higher risk of several adverse maternal–perinatal outcomes in singleton and twin pregnancies compared to non-GDM and non-HDP. Moreover, twin pregnancies complicated with GDM and HDP showed higher odds of adverse maternal–neonatal outcomes compared with singleton pregnancies complicated with GDM and HDP.

## Introduction

Gestational diabetes mellitus (GDM) and hypertensive disorders of pregnancy (HDP; including preeclampsia and gestational hypertension) are the most common pregnancy complications worldwide due to increasing advanced maternal age and higher prevalence of obesity in women of childbearing age (1, 2). These pregnancy complications are linked with short- and long-term adverse maternal–perinatal outcomes such as C-section, cardiovascular diseases, metabolic syndrome, premature delivery, low birth weight (LBW), perinatal mortality, intrauterine growth restriction (IUGR), macrosomia, neonatal shoulder dystocia, and neonatal respiratory morbidities (1–7). Over the last three decades, the trend of twin pregnancy rates has increased worldwide and in China (8, 9) and is associated with a higher risk of adverse pregnancy outcomes compared with singleton pregnancy (10, 11).

Twin pregnancies are associated with a higher risk of GDM and HDP compared with singleton pregnancies (12, 13). However, the findings on the association between GDM and HDP and maternal–neonatal outcomes among twin pregnancies are conflicting. Some studies reported that GDM and HDP are not associated with adverse maternal–neonatal outcomes among women with twins (2, 10, 14, 15). In contrast, others have found GDM and HDP to be associated with a higher risk of C-section and adverse perinatal outcomes (7, 13, 16–20). The conflicting findings could be attributed to the small sample size, lack of adjusting for important confounding factors, and lack of using singleton pregnancy as a control group in their studies (10, 14).

Very limited studies observed the adverse effect of GDM and HDP on maternal–perinatal outcomes in twin and singleton pregnancies (2, 7, 11, 21). These previous studies explored the association of GDM and HDP with adverse maternal–perinatal outcomes compared with non-GDM and non-HDP in singleton and twin pregnancies and failed to determine the risk of adverse pregnancy outcomes in twin pregnancies compared with singleton pregnancies complicated with GDM and HDP (2, 7, 11, 21). Moreover, some of these studies focused only on the association of GDM with adverse outcomes (11, 21), and others had small sample sizes (7) and included very limited adverse perinatal outcomes (2). It is rarely known that GDM and HDP may affect maternal–perinatal outcomes differentially among singleton and twin pregnancies. Therefore, our primary objective was to determine the adverse effect of GDM and HDP on maternal–perinatal outcomes among twin and singleton pregnancies compared to women without the aforementioned pregnancy complications. The secondary objective was to find the risk of adverse maternal–perinatal outcomes in twin pregnancies compared with singleton pregnancies complicated with GDM and HDP in Hubei, China.

## Materials and methods

### Study design and population

From 2011 to 2019, a retrospective-based study was conducted according to the Strengthening the Reporting of Observational Studies in Epidemiology (STROBE) guidelines (22) in the Department of Obstetrics and Gynecology at Wuhan University Renmin Hospital in Hubei, China. The data on pregnant women were collected and recorded during the individual examinations by the skilled nurses in the obstetrics register and electronic database. The study protocol was approved by the Ethical Review Board of Renmin Hospital (ID: WDRY2019–K034) in accordance with the Declaration of Helsinki.

### Inclusion and exclusion criteria and sample size calculation

**Figure 1:**
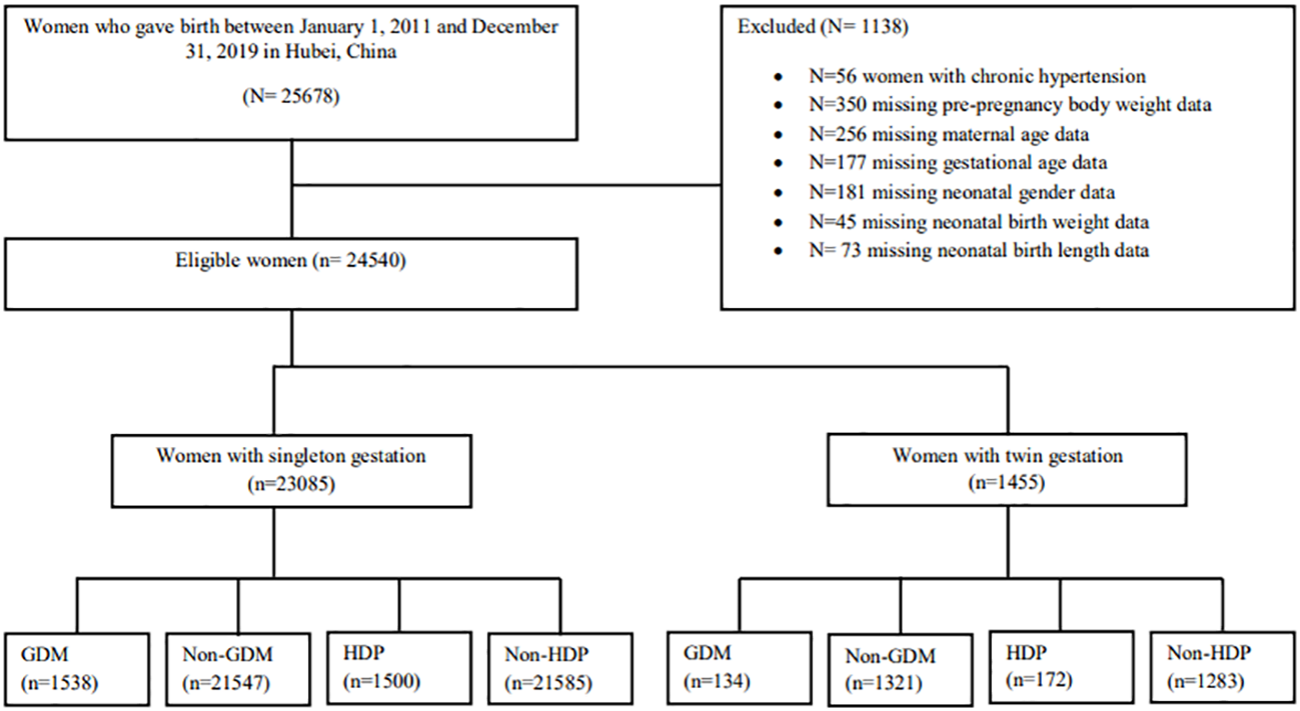
Flowchart of the study population.

A total of 24,540 pregnant women were included in the current study, including singleton (n = 23,085) and twin pregnancies (n = 1,455). The data on chronic hypertension (n = 56) and missing data on pre-pregnancy body weight (n = 350), maternal age (n = 256), gestational age (n = 177), neonatal gender (n = 181), birth weight (n = 45), and birth length (n = 73) were excluded from the statistical analysis as shown in **Figure 1**. The pattern of our missing data was missing at random (MAR), and for handling missing data MAR, the listwise or case deletion approach was applied, which simply means omitting subjects with the missing data and analyzing the remaining data (23). The sample size and power of the study were determined using the G*Power software (latest ver. 3.1.9.7; Heinrich-Heine-Universität Düsseldorf, Düsseldorf, Germany) (24). *A priori* power method was used by selecting Z tests (i.e., test family) and logistic regression (i.e., statistical test) to find the power analysis. It was assumed that 30% of non-GDM and non-HDP pregnant women had adverse maternal–perinatal outcomes and 50% of pregnant women with GDM and HDP had adverse maternal–perinatal outcomes. By considering the above assumptions and taking the power (1 − β = 0.80) and significance level (α = 0.05), the minimum sample size should be (N = 191).

### Collection of data on maternal traits

Data on maternal traits such as education, occupation, pre-pregnancy body weight, maternal age, gestational age, parity, and obstetric complications were collected from the obstetrics register. Maternal age was divided into two groups: i) <35 years and ii) ≥35 years at the time of delivery. The last known menstrual cycle date was used to determine gestational age, which was then verified by ultrasound examination in the first and second trimesters.

### Definition of exposure and outcome variables

The exposure variables were GDM and HDP (i.e., a composite of gestational hypertension, preeclampsia, and severe preeclampsia). GDM is defined as elevated blood sugar or glucose intolerance during pregnancy, which usually disappears after neonatal birth (18). Gestational hypertension (GH) is defined as having blood pressure greater than 140/90 mmHg without proteinuria after the 20th week of gestation (25). Preeclampsia (PE) is defined as elevated blood pressure 140/90 mmHg with proteinuria (albumin > 0.3 g in 24 hours) after the 20th week of gestation (26). Severe PE refers to having a blood pressure higher than 160/110 mmHg with proteinuria (albumin > 5 g in 24 hours) after the 20th week of gestation (27). The outcome variables are defined as follows. Placenta previa is defined as suboptimal placental implantation near or over the cervical opening (28). Placental abruption refers to the early separation of the placenta before childbirth (29). Neonatal birth outcomes were recorded immediately after neonatal birth including birth weight in grams using an electronic infant scale and birth length in centimeters using a standard measuring board for the neonate. Preterm birth is defined as a neonate born before 37 completed weeks or less than 259 days from the first date of a woman’s last menstrual period (30). Perinatal mortality is defined as the combination of late fetal mortality (stillbirths) and early neonatal mortality (0–6 days of life) (31). Fetal macrosomia is defined as birth weight ≥4,000 g, and LBW is defined as birth weight <2,500 g (32). IUGR is defined as a condition of fetal growth that is below the 10th percentile for its gestational age and does not reach its genetically predetermined growth potential (33). Apgar score was determined by evaluating the newborn baby on five simple criteria on a scale from zero to two and then summing up the five values obtained. Apgar score was recorded at 1 minute and 5 minutes after birth. Apgar score was divided into two categories: i) low Apgar score (<7) and ii) normal Apgar score (≥7) (34). Fetal hypoxia/distress is defined as a pathophysiological condition in which the fetus is suffering from insufficient oxygen supply (35). The ponderal index was determined by weight in g/(length in cm)^3^ × 100. A ponderal index between 2.5 and 3.0 was considered normal, between 2.0 and 2.5 marginal, and less than 2.0 low ponderal index (LPI) (36). A congenital defect is defined as an abnormality in the structure of neonatal body parts that occurs during intrauterine development (37).

### Definition of confounding factors

Confounding factors such as maternal age, education, occupation, pre-pregnancy body weight (≤45 kg and ≥91 kg), parity, and neonatal gender were selected based on previous literature, which is associated with both exposure and perinatal birth outcome (26).

### Statistical analysis

In the first step, a chi-square test was used for categorical and binary variables to find a significant difference in adverse maternal–perinatal outcomes between singleton and twin pregnancies. In the second step, multiple binary logistic regression models were used to find the effect of GDM and HDP on adverse maternal–perinatal outcomes among singleton and twin pregnancies compared with non-GDM and non-HDP groups. Moreover, we investigated the interaction effect of GDM (i.e., singleton × GDM and twins × GDM) and HDP (i.e., singleton × HDP and twins × HDP) on maternal–perinatal outcomes among singleton and twin pregnancies. In our analysis, the outcome variables were maternal outcomes (i.e., C-section, abnormal placentation, and premature rupture of membrane (PROM)) and neonatal outcomes (i.e., preterm births, perinatal mortality, LBW, IUGR, and congenital defects). The exposure variables such as GDM and HDP were taken as predictor variables. In the third step, our analysis was restricted to singleton pregnancies complicated with GDM (n = 1,538) and HDP (n = 1,500) and twin pregnancies complicated with GDM (n = 134) and HDP (n = 172). The adjusted odds ratio (aOR) of adverse maternal–perinatal outcomes in twin pregnancies compared with singleton pregnancies complicated with GDM and HDP was determined. Here, twin pregnancies were taken as the predictor variable (using singleton pregnancies as reference), and maternal–perinatal outcomes were the outcomes variables. The multiple binary logistic regression models were adjusted for confounding factors as previously defined. Adjusted odds ratios with 95% confidence intervals were used to estimate the association between predictor variables and outcome variables. *p*-Value (two-tailed <0.05) was taken as statistically significant. The data were analyzed using Statistical Package for Social Sciences (SPSS) for Windows version 22 (IBM Corporation, New York, NY, USA).

In the fourth step, joinpoint regression analysis was used to estimate the temporal trend of GDM and HDP among singleton and twin pregnancies during the study period (2011–2019). The annual percentage changes (APCs) and average annual percentage changes (AAPCs) in the rate of GDM and HDP were estimated for each segment/period in the regression analysis. The AAPC represents the trend in GDM and HDP in the whole period 2011–2019, while the APC indicates the trend in GDM and HDP in each segment/period identified by the joinpoint regression software. The temporal trend is considered positive when the AAPC or APC >0 with its 95% confidence interval (CI); however, the AAPC or APC <0 with its 95%CI shows a negative trend. Moreover, Monte Carlo methods were used to find each *p*-value and maintain the overall asymptotic significance level through Bonferroni correction. This analysis was conducted using the joinpoint regression program version 4.8.0.1 (April 2020) from the Surveillance Research Program of the U.S. National Cancer Institute.

## Results

### Maternal–perinatal characteristics among twin vs. singleton pregnancies

**Figure 2:**
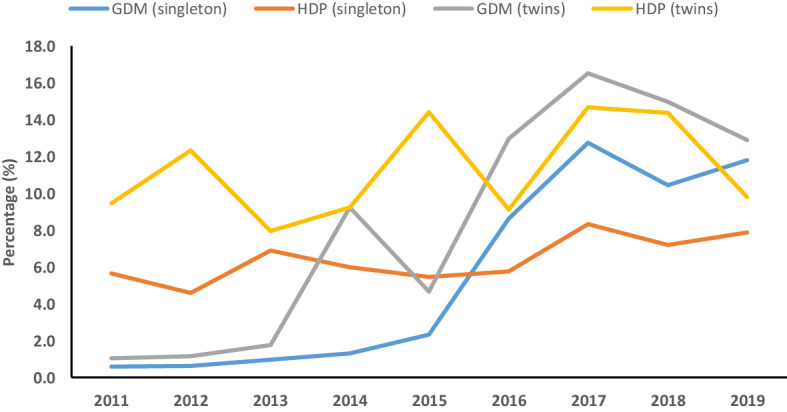
Temporal trend of gestational diabetes mellitus (GDM) and hypertensive disorders of pregnancy (HDP) among singleton and twin pregnancies (2011–2019).

Out of 25,678 pregnant women, 24,540 women met the inclusion criteria in the current study including singleton (n = 23,085) and twin pregnancies (n = 1,455) (**Figure 1**). Twin pregnancies showed a significantly higher prevalence of C-section (80.8% vs. 60.7%), GDM (9.2% vs. 6.7%), HDP (11.8% vs. 6.5%), preterm birth (PTB) (72.8% vs. 19.2%), perinatal mortality (2.3% vs. 1.4%), LBW (64.8% vs. 14.2%), LPI (9.0% vs. 3.9%), and low Apgar score (15.7% vs. 3.7%) compared with singleton pregnancies (*p* < 0.05) (**Figure 2**). However, singleton pregnancies had a statistically significantly higher prevalence of oligohydramnios (3.5% vs. 0.6%), fetal distress (2.3% vs. 1.2%), macrosomia (5.4% vs. 0.1%), and congenital defects (1.3% vs. 0.5%) compared with twin pregnancies (**Tables 1**, **2**). Moreover, we compared the maternal–perinatal outcomes of missing and excluded women (n = 1,138) with those included in the current study and showed similar findings (data not shown).

**Table 1:** Distribution of maternal characteristics and pregnancy complications among twin vs. singleton pregnancies (N = 24,540).

Maternal traits and pregnancy complications	Singleton (n = 23,085)	Twins (n = 1,455)	*p*-Value
	No. %	No. %	
Maternal education			
Low	4,953 (21.5)	389 (26.7)	0.001
Middle	9,086 (39.4)	557 (38.3)	
Higher	9,046 (39.2)	509 (35.0)	
Maternal occupation			
Housewives	12,065 (52.3)	819 (56.3)	0.01
Professional services	10,472 (45.4)	603 (41.4)	
Manual workers	548 (2.4)	33 (2.3)	
AMA^*^	3,956 (17.1)	265 (18.2)	0.2
Parity			
Primiparous (≤1)	17,514 (75.9)	1,170 (80.5)	0.001
Multiparous (>1)	5,571 (24.1)	284 (19.5)	
C-section^*^	14,019 (60.7)	1,175 (80.8)	0.001
Previous history of C-section^*^	3,595 (15.6)	155 (10.7)	0.001
GDM^*^	1,538 (6.7)	134 (9.2)	0.001
HDP^*^	1,500 (6.5)	172 (11.8)	0.001
Abnormal Placentation^*^	1,017 (4.4)	39 (2.7)	0.002
PROM^*^	2,153 (9.3)	170 (11.7)	0.003
Fetal breech presentation^*^	575 (2.5)	40 (2.7)	0.5
Oligohydramnios^*^	805 (3.5)	9 (0.6)	0.001
Polyhydramnios^*^	91 (0.4)	9 (0.6)	0.1
Nuchal cord^*^	996 (4.3)	77 (5.3)	0.08

p-Values were calculated using chi-square test.

* Frequency and percentage of variables with only “yes” value presented; AMA, advanced maternal age ≥35 years); HDP, hypertensive disorders of pregnancy; GDM, gestational diabetes mellitus; PROM, premature rupture of membrane.

**Table 2:** Distribution of perinatal outcomes among twin vs. singleton pregnancies.

Perinatal outcomes	Singleton (n = 23,085)	Twins (n = 1,455)	*p*-Value
	No. %	No. %	
PTB^*^	4,430 (19.2)	1,059 (72.8)	0.001
Perinatal mortality*	333 (1.4)	33 (2.3)	0.01
LBW*	3,283 (14.2)	943 (64.8)	0.001
IUGR*	168 (0.7)	6 (0.4)	0.1
LPI*	898 (3.9)	131 (9.0)	0.001
Low Apgar score*	844 (3.7)	229 (15.7)	0.001
Fetal distress*	522 (2.3)	18 (1.2)	0.01
Macrosomia*	1,252 (5.4)	2 (0.1)	0.001
Congenital defects*	298 (1.3)	8 (0.5)	0.01
Neonatal gender			
Male	12,340 (53.5)	764 (52.5)	0.4
Female	10,745 (46.5)	691 (47.5)	

p-values were calculated using chi-square test.

* Frequency and percentage of variables with only “yes” value presented; PTB, preterm birth; LBW, low birth weight; IUGR, intrauterine growth restriction; LPI, low ponderal index; *congenital defects (microtia, anotia, polydactyly, heart defects, limb reduction defects, cleft lip, cleft palate, hydrocephaly, and neural tube defects (NTDs)).

### Maternal–perinatal outcomes between GDM and non-GDM singleton and twin pregnancies

Among singleton pregnancies, after adjusting for confounding factors, the risk of C-section (aOR, 1.5; 95%CI: 1.3, 1.6), polyhydramnios (aOR, 1.9; 95%CI: 1.1, 3.7), and macrosomia (aOR, 1.3; 95%CI: 1.1, 1.6) was significantly higher in the GDM group compared with the non-GDM group. Moreover, among twin pregnancies, the risk of polyhydramnios (aOR, 5.4; 95%CI: 1.3, 22.7) and PTB (aOR, 2.1; 95%CI: 1.2, 3.3) was significantly higher in the GDM group compared with the non-GDM group. Compared with non-GDM, the interaction effect between twins and GDM (i.e., twins × GDM) markedly increased the risk of C-section (aOR, 2.2; 95%CI: 1.5, 3.5), PTB (aOR, 18.9; 95%CI: 11.9, 30.0), LBW (aOR, 8.1; 95%CI: 5.7, 11.5), and low Apgar score (aOR, 4.3; 95%CI: 2.6, 7.1) (**Tables 3**, **4**).

**Table 3:** Maternal–perinatal outcomes between GDM and non-GDM singleton pregnancies.

Maternal–neonatal outcomes	Singleton			Interaction effect
	Non-GDM (N = 21,547)	GDM (N = 1,538)	aOR (95%CI)	Singleton × GDM aOR (95%CI)
C-section	12,929 (60.0)	1,090 (70.9)	1.5 (1.3, 1.6)	1.3 (1.2, 1.5)*
Abnormal placentation	955 (4.4)	62 (4.0)	0.8 (0.6, 1.1)	0.8 (0.6, 1.1)*
PROM	2,049 (9.5)	104 (6.8)	0.7 (0.5, 0.8)	0.7 (0.5, 0.8)*
Fetal breech presentation	538 (2.5)	37 (2.4)	0.9 (0.6, 1.3)	0.9 (0.6, 1.3)*
Oligohydramnios	763 (3.5)	42 (2.7)	0.7 (0.5, 1.0)	0.8 (0.6, 1.1)*
Polyhydramnios	80 (0.4)	11 (0.7)	1.9 (1.1, 3.7)	1.8 (1.1, 3.5)**
Nuchal cord	946 (4.4)	50 (3.3)	0.7 (0.5, 1.0)	0.7 (0.5, 1.0)*
PTB	4,123 (19.1)	307 (20.0)	1.1 (0.8, 1.1)	0.8 (0.7, 0.9)*
Perinatal mortality	313 (1.5)	20 (1.3)	0.8 (0.5, 1.3)	0.8 (0.5, 1.3) *
LBW	3,094 (14.4)	189 (12.3)	0.8 (0.7, 0.9)	0.6 (0.5, 0.7)*
IUGR	158 (0.7)	10 (0.7)	0.9 (0.4, 1.7)	0.9 (0.4, 1.7) **
LPI	844 (3.9)	54 (3.5)	0.9 (0.6, 1.1)	0.8 (0.6, 1.1) *
Low Apgar score	809 (3.8)	35 (2.3)	0.5 (0.4, 0.8)	0.5 (0.3, 0.7) *
Fetal distress	498 (2.3)	24 (1.6)	0.6 (0.4, 1.1)	0.6 (0.4, 1.1) *
Macrosomia	1,141 (5.3)	111 (7.2)	1.3 (1.1, 1.6)	1.4 (1.1, 1.7) *
Congenital defects	280 (1.3)	18 (1.2)	0.9 (0.5, 1.4)	0.9 (0.6, 1.5) *

GDM, gestational diabetes mellitus; PROM, premature rupture of membrane; PTB, preterm birth; LBW, low birth weight; IUGR, intrauterine growth restriction; LPI, low ponderal index; aOR, adjusted odds ratios; CI, confidence interval. Adjusted for parity, maternal age, education, occupation, pre-pregnancy body weight, and neonatal gender; non-GDM was taken as a reference group. * Statistical power = 80%, ** statistical power < 80%.

**Table 4:** Maternal–perinatal outcomes between GDM and non-GDM twin pregnancies.

Maternal–neonatal outcomes	Twins			Interaction effect
	Non-GDM (N = 1,321)	GDM (N = 134)	aOR (95%CI)	Twins × GDM aOR (95%CI)
C-section	1,066 (80.8)	109 (81)	0.9 (0.6, 1.5)	2.2 (1.5, 3.5) *
Abnormal placentation	36 (2.7)	3 (2.2)	0.8 (0.2, 2.7)	0.5 (0.1, 1.6) **
PROM	159 (12.1)	11 (8.2)	0.6 (0.3, 1.2)	0.8 (0.4, 1.6) **
Fetal breech presentation	39 (3.0)	2 (1.4)	0.2 (0.03, 1.8)	0.2 (0.04, 2.1) **
Oligohydramnios	7 (0.5)	2 (1.4)	2.6 (0.5, 13.4)	0.4 (0.1, 1.7) **
Polyhydramnios	6 (0.4)	3 (2.2)	5.4 (1.3, 22.7)	6.1 (1.8, 19.3) **
Nuchal cord	68 (5.2)	9 (6.7)	1.3 (0.6, 2.8)	1.6 (0.8, 3.2) **
PTB	946 (71.7)	112 (83.6)	2.1 (1.2, 3.3)	18.9 (11.9, 30.0) *
Perinatal mortality	29 (2.2)	4 (3.0)	1.3 (0.4, 3.8)	2.2 (0.8, 6.1) **
LBW	862 (65.3)	81 (60.4)	0.7 (0.5, 1.1)	8.1 (5.7, 11.5) *
IUGR	5 (0.4)	1 (0.7)	2.3 (0.2, 20.1)	1.1 (0.1, 8.2) **
LPI	123 (9.3)	8 (6.0)	0.6 (0.2, 1.3)	1.5 (0.7, 3.2) **
Low Apgar score	209 (15.8)	20 (14.9)	0.9 (0.5, 1.5)	4.3 (2.6, 7.1) **
Fetal distress	16 (1.2)	2 (1.4)	1.4 (0.3, 6.3)	0.6 (0.2, 2.7) **
Macrosomia	2 (0.2)	0 (0)	0 (0, 0)	0 (0, 0)
Congenital defects	8 (0.6)	0 (0)	0 (0, 0)	0 (0, 0)

GDM, gestational diabetes mellitus; PROM, premature rupture of membrane; PTB, preterm birth; LBW, low birth weight; IUGR, intrauterine growth restriction; LPI, low ponderal index; aOR, adjusted odds ratios; CI, confidence interval. Adjusted for parity, maternal age, education, occupation, pre-pregnancy body weight, and neonatal gender; non-GDM was taken as a reference group. * Statistical power = 80%, ** statistical power < 80%.

### Maternal–perinatal outcomes between HDP and non-HDP singleton and twin pregnancies

Among singleton pregnancies, the risk of C-section (aOR, 1.9; 95%CI: 1.7, 2.1), PTB (aOR, 3.1; 95%CI: 2.7, 3.3), perinatal mortality (aOR, 1.8; 95%CI: 1.3, 2.5), LBW (aOR, 3.9; 95%CI: 3.4, 4.3), IUGR (aOR, 3.5; 95%CI: 2.4, 5.2), LPI (aOR, 2.8; 95%CI: 2.3, 3.4), and low Apgar score (aOR, 2.4; 95%CI:1.9, 2.9) was significantly higher in the HDP group compared with the non-HDP group. Among twin pregnancies, the risk of C-section (aOR, 3.5; 95%CI: 1.8, 6.6), PTB (aOR, 2.1; 95%CI: 1.3, 3.1), perinatal mortality (aOR, 2.9; 95%CI: 1.2, 6.6), LBW (aOR, 1.6; 95%CI: 1.1, 2.3), LPI (aOR, 1.6; 95%CI: 1.0, 2.6), and low Apgar score (aOR, 1.5; 95%CI: 1.0, 2.3) was significantly higher in the HDP group compared with the non-HDP group. Moreover, the interaction effect between twins and HDP (i.e., twins × HDP) remarkably increased the odds of C-section (aOR, 8.5; 95%CI: 4.6, 15.8), PTB (aOR, 19.1; 95%CI: 12.7, 28.5), perinatal mortality (aOR, 3.8; 95%CI: 1.8, 8.1), LBW (aOR, 14.4; 95%CI: 10.2, 20.3), LPI (aOR, 3.7; 95%CI: 2.4, 5.9), and low Apgar score (aOR, 7.1; 95%CI: 4.8, 10.4) compared with non-HDP (**Tables 5**, **6**).

**Table 5:** Maternal–perinatal outcomes between HDP and non-HDP singleton pregnancies.

Maternal–neonatal outcomes	Singleton			Interaction effect
	Non-HDP (N = 21,585)	HDP (N = 1,500)	aOR (95%CI)	Singleton × HDP aOR (95%CI)
C-section	12,891 (59.7)	1,128 (75.2)	1.9 (1.7, 2.1)	1.8 (1.5, 2.0)*
Abnormal placentation	969 (4.5)	48 (3.2)	0.6 (0.4, 0.8)	0.6 (0.4, 0.8)*
PROM	2,110 (9.8)	43 (2.9)	0.2 (0.2, 0.3)	0.2 (0.2, 0.3)*
Fetal breech presentation	544 (2.5)	31 (2.1)	0.8 (0.5, 1.1)	0.7 (0.5, 1.1)*
Oligohydramnios	765 (3.5)	40 (2.7)	0.7 (0.5, 1.1)	0.8 (0.5, 1.1)*
Polyhydramnios	88 (0.4)	3 (0.2)	0.4 (0.1, 1.5)	0.4 (0.2, 1.4)**
Nuchal cord	960 (4.4)	36 (2.4)	0.5 (0.3, 0.7)	0.5 (0.3, 0.7)*
PTB	3,829 (17.7)	601 (40.1)	3.1 (2.7, 3.3)	2.3 (2.1, 2.6)*
Perinatal mortality	294 (1.4)	39 (2.6)	1.8 (1.3, 2.5)	1.7 (1.2, 2.5)*
LBW	2,738 (12.7)	545 (36.3)	3.9 (3.4, 4.3)	2.9 (2.6, 3.2)*
IUGR	134 (0.6)	34 (2.3)	3.5 (2.4, 5.2)	3.5 (2.4, 5.2)**
LPI	758 (3.5)	140 (9.3)	2.8 (2.3, 3.4)	2.5 (2.1, 3.1)*
Low Apgar score	726 (3.4)	118 (7.9)	2.4 (1.9, 2.9)	1.9 (1.5, 2.3)*
Fetal distress	495 (2.3)	27 (1.8)	0.7 (0.5, 1.1)	0.8 (0.5, 1.2)*
Macrosomia	1,187 (5.5)	65 (4.3)	0.7 (0.5, 0.9)	0.8 (0.6, 1.0)*
Congenital defects	283 (1.3)	15 (1.0)	0.7 (0.4, 1.2)	0.7 (0.4, 1.3)*

HDP, hypertensive disorders of pregnancy; PROM, premature rupture of membrane; PTB, preterm birth; LBW, low birth weight; IUGR, intrauterine growth restriction; LPI, low ponderal index; aOR, adjusted odds ratios; CI, confidence interval. Adjusted for parity, maternal age, education, occupation, pre-pregnancy body weight, and neonatal gender; non-HDP was taken as a reference group. * Statistical power = 80%, ** statistical power < 80%.

**Table 6:** Maternal–perinatal outcomes between HDP and non-HDP twin pregnancies.

Maternal–neonatal outcomes	Twins			Interaction effect
	Non-HDP (N = 1,283)	HDP (N = 172)	aOR (95%CI)	Twins × HDP aOR (95%CI)
C-section	1,014 (79.0)	161 (93.6)	3.5 (1.8, 6.6)	8.5 (4.6, 15.8)*
Abnormal Placentation	38 (3.0)	1 (0.6)	0.2 (0.1, 1.4)	0.1 (0.1, 0.9)**
PROM	162 (12.6)	8 (4.7)	0.3 (0.1, 0.7)	0.4 (0.2, 0.9)**
Fetal breech presentation	37 (2.9)	3 (1.7)	0.5 (0.1, 1.8)	0.6 (0.2, 2.1)**
Oligohydramnios	9 (0.7)	0 (0)	0 (0, 0)	0 (0, 0)
Polyhydramnios	8 (0.8)	1 (0.6)	1.1 (0.1, 8.8)	1.5 (0.2, 11.2)**
Nuchal cord	67 (5.2)	10 (5.8)	1.1 (0.5, 2.2)	1.4 (0.7, 2.7)**
PTB	916 (71.4)	143 (83.1)	2.1 (1.3, 3.1)	19.1 (12.7, 28.5)*
Perinatal mortality	25 (1.9)	8 (4.7)	2.9 (1.2, 6.6)	3.8 (1.8, 8.1)**
LBW	819 (63.8)	124 (72.1)	1.6 (1.1, 2.3)	14.4 (10.2, 20.3)*
IUGR	6 (0.5)	0 (0)	0 (0, 0)	0 (0, 0)
LPI	109 (8.5)	22 (12.8)	1.6 (1.0, 2.6)	3.7 (2.4, 5.9)**
Low Apgar score	192 (15.0)	37 (21.5)	1.5 (1.0, 2.3)	7.1 (4.8, 10.4)*
Fetal distress	16 (1.2)	2 (1.2)	1.2 (0.2, 5.5)	0.5 (0.1, 2.1)**
Macrosomia	2 (0.2)	0 (0)	0 (0, 0)	0 (0, 0)
Congenital defects	8 (0.6)	0 (0)	0 (0, 0)	0 (0, 0)

HDP, hypertensive disorders of pregnancy; PROM, premature rupture of membrane; PTB, preterm birth; LBW, low birth weight; IUGR, intrauterine growth restriction; LPI, low ponderal index; aOR, adjusted odds ratios; CI, confidence interval. Adjusted for parity, maternal age, education, occupation, pre-pregnancy body weight, and neonatal gender; non-HDP taken as a reference group. * Statistical power = 80%, ** statistical power < 80%.

### Risk of adverse outcomes in twin pregnancies complicated with GDM and HDP compared with singleton pregnancies complicated with GDM and HDP

After adjusting for confounding factors, twin pregnancies complicated with GDM and HDP showed higher odds of C-section [(aOR, 1.7; 95%CI: 1.1, 2.7), (aOR, 4.6; 95%CI: 2.5, 8.7), respectively], nuchal cord [(aOR, 2.4; 95%CI: 1.2, 5.1), (aOR, 2.3; 95%CI: 1.1, 4.7), respectively], PTB [(aOR, 22.9; 95%CI: 14.1, 37.3), (aOR, 8.1; 95%CI: 5.3, 12.3), respectively], LBW [(aOR, 12.1; 95%CI: 8.2, 18.1), (aOR, 5.1; 95%CI: 3.6, 7.4), respectively], and low Apgar score [(aOR, 8.2; 95%CI: 4.4, 15.1), (aOR, 3.8; 95%CI: 2.4, 5.8), respectively] compared with singleton pregnancies complicated with GDM and HDP (**Table 7**).

**Table 7:** Maternal–perinatal outcomes in twin pregnancies complicated with GDM and HDP compared with singleton pregnancies complicated with GDM and HDP. .

Maternal–neonatal outcomes	Singleton aOR (95%CI)	Twins	
		GDM [aOR (95%CI)]	HDP [aOR (95%CI)]
C-section	1.00 (reference)	1.7 (1.1, 2.7)*	4.6 (2.5, 8.7)*
Abnormal placentation	1.00 (reference)	0.6 (0.1, 2.1)**	0.2 (0.1, 1.5)**
PROM	1.00 (reference)	1.2 (0.6, 2.4)**	1.7 (0.7, 3.8)**
Fetal breech presentation	1.00 (reference)	0.3 (0.04, 2.2)**	0.8 (0.2, 2.8)**
Oligohydramnios	1.00 (reference)	0.4 (0.1, 2.1)**	0 (0, 0)
Polyhydramnios	1.00 (reference)	3.5 (0.9, 13.2)**	5.3 (0.4, 58)**
Nuchal cord	1.00 (reference)	2.4 (1.2, 5.1)**	2.3 (1.1, 4.7)**
PTB	1.00 (reference)	22.9 (14.1, 37.3)*	8.1 (5.3, 12.3)*
Perinatal mortality	1.00 (reference)	2.6 (0.8, 8.1)**	2.3 (1.1, 5.1)**
LBW	1.00 (reference)	12.1 (8.2, 18.1)*	5.1 (3.6, 7.4)*
IUGR	1.00 (reference)	1.1 (0.1, 8.0)**	0 (0, 0)
LPI	1.00 (reference)	1.7 (0.7, 3.7)**	1.4 (0.9, 2.4)**
Low Apgar score	1.00 (reference)	8.2 (4.4, 15.1)**	3.8 (2.4, 5.8)**
Fetal distress	1.00 (reference)	0.9 (0.2, 4.0)**	0.6 (0.1, 2.7)**
Macrosomia	1.00 (reference)	0 (0, 0)	0 (0, 0)
Congenital defects	1.00 (reference)	0 (0, 0)	0 (0, 0)

GDM, gestational diabetes mellitus; HDP, hypertensive disorders of pregnancy; PROM, premature rupture of membrane; PTB, preterm birth; LBW, low birth weight; IUGR, intrauterine growth restriction; LPI, low ponderal index; aOR, adjusted odds ratios; CI, confidence interval. Adjusted for parity, maternal age, education, occupation, pre-pregnancy body weight, neonatal gender, twin pregnancies complicated with GDM (n = 134) and HDP (n = 172), and singleton pregnancies complicated with GDM (n = 1,538) and HDP (n = 1,500). * Statistical power = 80%, ** statistical power < 80%.

### Temporal trend of GDM and HDP in singleton and twin pregnancies (2011–2019)

The joinpoint regression analysis showed that the APC of GDM significantly increased among singleton (APC, 95.1%; 95%CI: 11.9, 240) and twin pregnancies (APC, 86.3%; 95%CI: 53.5, 126) during 2011–2017. Moreover, the trend of HDP (AAPC, 15.1%; 95%CI: 5.3, 25.7) among singleton pregnancies and GDM (AAPC, 50.4%; 95%CI: 19.9, 88.7) among twin pregnancies significantly increased from 2011 to 2019 (**Table 8**, **Figure 2**).

**Table 8:** Temporal trend of GDM and HDP in singleton and twin pregnancies using joinpoint regression analysis (2011–2019).

Variables	Singleton		Twins	
	Year	APC (95%CI)	Year	APC (95%CI)
GDM				
Trend1	2011–2017	95.1 (11.9, 240)	2011–2017	86.3 (53.5, 126)
Trend2	2017–2019	4.3 (−96.1, 2695)	2017–2019	−20.9 (−74.8, 148)
AAPC (95%CI)	2011–2019	66.9 (−12.9, 219)	2011–2019	50.4 (19.9, 88.7)
HDP				
Trend1	2011–2014	−0.5 (−23.9, 30.1)	2011–2017	19.7 (−26.7, 95.3)
Trend2	2014–2019	25.5 (11.3, 41.5)	2017–2019	−13.0 (−95.2, 1475)
AAPC (95%CI)	2011–2019	15.1 (5.3, 25.7)	2011–2019	10.5 (−37.7, 96.0)

GDM, gestational diabetes mellitus; HDP, hypertensive disorders of pregnancy; APC, annual percentage change; AAPC, average annual percent change; CI, confidence interval.

## Discussion

In the present study, we observed a significantly higher prevalence of GDM, HDP, and adverse neonatal outcomes among twins compared with singleton pregnancies. GDM increased the risk of C-section and macrosomia in singleton pregnancies and PTB in twin pregnancies compared with non-GDM. In both the singleton and twin pregnancies, HDP was associated with a higher risk of C-section, PTB, perinatal mortality, LBW, LPI, and low Apgar score compared with non-HDP. Moreover, twin pregnancies complicated with GDM and HDP showed higher odds of adverse maternal–neonatal outcomes compared with singleton pregnancies. The temporal trend of HDP among singleton pregnancies and GDM among twin pregnancies significantly increased from 2011 to 2019.

### Adverse maternal–perinatal outcomes among twin vs. singleton pregnancies

We found that twin pregnancies showed a higher prevalence of GDM and HDP compared with singleton pregnancies, which is supported by several previous findings (2, 11, 18, 38). It could be due to the elevated levels of placental hormones including estrogen, lactogen, and progesterone in twin pregnancies compared with singleton pregnancies, which may cause a higher prevalence of GDM due to their insulin-antagonistic effects. Moreover, twin pregnancies had greater placental mass compared with singleton pregnancies, and a higher prevalence of both HDP and GDM is associated with greater placental mass (18, 39–41). In our study, the higher prevalence of C-section, PTB, perinatal mortality, LBW, LPI, and low Apgar score in twin pregnancies compared with singleton pregnancies could be attributed to the higher incidence of GDM and HDP. Several lines of evidence show that GDM and HDP are associated with a higher prevalence of adverse maternal–neonatal outcomes (1, 2, 18, 41).

### Maternal–perinatal outcomes between GDM and non-GDM singleton and twin pregnancies

We observed that GDM increased the risk of macrosomia in singleton pregnancies compared with non-GDM. The higher odds of macrosomia in singleton pregnancies could be due to the exposure of neonates to higher glucose levels over a prolonged gestational period (42). We found that singleton women had a significantly higher prevalence of full-term pregnancies compared with twin women. Moreover, it is well established that GDM pregnancies had a higher risk of accelerated fetal growth in singleton pregnancies (43–45). However, in twin pregnancies, no macrosomia was observed in the GDM group perhaps due to a higher prevalence of premature delivery compared with singleton pregnancies, which is consistent with previous findings (1, 46). GDM was associated with higher odds of PTB in twins but not in singleton pregnancies. Gonzalez et al. (47) also observed that GDM was associated with a higher risk of premature birth in twin pregnancies. However, Hiersch et al. (11) reported that GDM was associated with an increased risk of PTB in both singleton and twin pregnancies. However, some previous studies showed no risk of PTB among GDM-twin pregnancies (1, 14, 17). The variation in these findings could be due to different population sample sizes (1, 11).

### Maternal–perinatal outcomes between HDP and non-HDP singleton and twin pregnancies

We found that in both singleton and twin pregnancies, HDP was associated with a higher risk of C-section, PTB, perinatal mortality, LBW, LPI, and low Apgar score compared with non-HDP. Twin pregnancies showed a higher prevalence of HDP compared with singleton pregnancies, but HDP had more severe adverse effects on perinatal outcomes including PTB, LBW, LPI, IUGR, and low Apgar score in singleton pregnancies except for perinatal mortality. This higher adverse effect on perinatal outcomes could be related to the severity and earlier onset of HDP (including preeclampsia and gestational hypertension) in singleton pregnancies. In contrast, Sibai et al. (13) found that twin pregnancies complicated with gestational hypertension and preeclampsia had more adverse effects on PTB, small for gestational age (SGA), and lower neonatal birth weight than singleton pregnancies. A series of previous studies also observed a higher risk of adverse neonatal outcomes in twin pregnancies complicated with HDP compared with singleton pregnancies (7, 48). The contradiction in these findings is mainly due to considering different reference groups. In our analysis, the reference group for adverse pregnancy outcomes was a non-HDP group for both singleton and twin pregnancies. However, previous studies failed to consider the non-HDP group as a reference group for adverse pregnancy outcomes in singleton and twin gestations (7, 13, 48).

### Risk of adverse outcomes in twin pregnancies complicated with GDM and HDP compared with singleton pregnancies complicated with GDM and HDP

We restricted our analysis to GDM and HDP pregnant women and compared the risk of adverse pregnancy outcomes in twin pregnancies with singleton pregnancies. We found that twins complicated with GDM and HDP showed higher odds of C-section, nuchal cord, PTB, LBW, and low Apgar score than singleton pregnancies in a reference group. However, GDM had a more severe adverse effect on neonatal outcomes, particularly on PTB, LBW, and low Apgar score than HDP in twin pregnancies. It indicates that GDM and HDP may differently affect neonatal outcomes in twins, suggesting a need for further studies to evaluate the different effects of GDM and HDP on perinatal outcomes in twin pregnancies. Luo et al. (21) observed that diabetes in twin pregnancies was associated with an increased risk of macrosomia, PTB, and congenital defects but a reduced risk of low Apgar scores and neonatal deaths compared with singleton pregnancies. Foo et al. (7) investigated the impact of HDP on pregnancy outcomes in twin versus singleton pregnancies. They found that twins showed a higher risk of preterm birth and low birth weight but that there was no significant difference in perinatal mortality between twins and singletons complicated with HDP. However, we found that twin women had a higher risk of perinatal mortality than singleton associated with HDP. The variation in these findings could be due to a small sample size (7) and the use of different reference groups for adverse pregnancy outcomes (21). In the previous study (21), they used non-GDM women as a reference group; however, we used singleton as a reference group for adverse pregnancy outcomes in twins.

### Temporal trend of GDM and HDP in singleton and twin pregnancies

We observed that the temporal trend of HDP among singleton pregnancies and GDM among twin pregnancies significantly increased during the study period (2011 to 2019). The increasing trend in GDM and HDP could be due to an increase in advanced maternal age, a higher prevalence of obesity in women of childbearing age, and universal screening for GDM and HDP (1, 2). In Tianjin China, the prevalence of GDM increased from 2.3% in 1999 (49) to 9.3% in 2012 (50). In Xiamen Fujian, the trend of GDM increased by 28% from 2012 to 2017 (51). A higher prevalence of HDP was observed in Western and Northern China (52). The increasing trend of GDM and HDP in Chinese women could be due to advanced maternal age, pre-pregnancy higher body mass index (BMI), higher gestational weight gain, smoking, urbanization, and lifestyle changes (50–52). However, we did not find the risk factors associated with the increasing trend of GDM and HDP in our study due to the lack of certain data such as BMI, smoking, and gestational weight gain.

Our study had several limitations. First, our findings are based on a retrospective study. Second, our findings are from a single-center tertiary hospital, which could be a potential selection bias in our study. Third, our study had missing data (i.e., 4%) and lacked certain data including maternal obesity, gestational weight gain, smoking and alcohol habits, and assisted reproductive technology. To address the issue of missing data, we compared the outcomes of excluded women with those included in the current study, and they showed similar findings (data not shown). Fourth, due to the small sample size, the results cannot be generalized to the overall population.

## Conclusion

In conclusion, twin pregnancies showed a significantly higher prevalence of GDM, HDP, and certain adverse perinatal outcomes than singleton pregnancies. GDM is associated with higher odds of C-section and macrosomia in singleton and with higher odds of PTB in twin pregnancies compared with non-GDM. In both singleton and twin pregnancies, HDP was associated with a higher risk of several adverse maternal–neonatal outcomes compared with non-HDP. Furthermore, twin pregnancies complicated with GDM and HDP showed higher odds of adverse maternal–neonatal outcomes compared with singleton pregnancies complicated with GDM and HDP. The temporal trend of HDP among singleton pregnancies and GDM among twin pregnancies significantly increased during the study period. These findings can be used to provide meaningful information for gynecologists and clinicians to timely identify and manage both singleton and twin pregnancies complicated with GDM and HDP.

## Data availability statement

The original contributions presented in the study are included in the article/supplementary material. Further inquiries can be directed to the corresponding authors.

## Ethics statement

The studies involving humans were approved by the Ethical Review Board of Renmin Hospital (ID: WDRY2019–K034) in accordance with the Declaration of Helsinki. The studies were conducted in accordance with the local legislation and institutional requirements. The ethics committee/institutional review board waived the requirement of written informed consent for participation from the participants or the participants’ legal guardians/next of kin because it was a retrospective study.

## Author contributions

N: Conceptualization, Data curation, Formal analysis, Funding acquisition, Methodology, Resources, Software, Visualization, Writing – original draft, Writing – review & editing. ZLe:Conceptualization, Data curation, Writing – review & editing. ZYL: Data curation, Writing – review & editing. SM: Data curation, Formal analysis, Methodology, Writing – review & editing, Visualization. YS: Data curation, Methodology, Validation, Writing – review & editing, Visualization. SN: Data curation, Methodology, Validation, Writing – review & editing. HL: Conceptualization, Investigation, Project administration, Supervision, Validation, Visualization, Writing – review & editing.
